# Sleep Trajectories of Women Undergoing Elective Cesarean Section: Effects on Body Weight and Psychological Well-Being

**DOI:** 10.1371/journal.pone.0129094

**Published:** 2015-06-12

**Authors:** Ya-Ling Tzeng, Shu-Ling Chen, Chuen-Fei Chen, Fong-Chen Wang, Shu-Yu Kuo

**Affiliations:** 1 School of Nursing, China Medical University and China Medical University Hospital, Taichung, Taiwan; 2 Department of Nursing, Hungkuang University, Taichung, Taiwan; 3 Department of Medicine, Mackay Medical College, New Taipei City, Taiwan; 4 School of Nursing, College of Nursing, Taipei Medical University, Taipei, Taiwan; University of Rome Tor Vergata, ITALY

## Abstract

**Background:**

After cesarean section (CS), women may be at great risk for sleep disturbance, but little is known about temporal changes in their sleep patterns and characteristics. We had two aims: 1) to identify distinct classes of sleep-disturbance trajectories in women considering elective CS from third-trimester pregnancy to 6 months post-CS and 2) to examine associations of sleep trajectories with body mass index (BMI), depressive symptoms, and fatigue scores.

**Methods:**

We analyzed data from a prospective cohort study of 139 Taiwanese pregnant women who elected CS. Sleep components were assessed using the Pittsburgh Sleep Quality Index in third-trimester pregnancy, 1 day, 1 week, 1 month, and 6 months post-CS. Data were collected on depressive symptoms, fatigue symptoms, and BMI. Sleep-quality trajectories were identified by group-based trajectory modeling.

**Results:**

We identified three distinct trajectories: stable poor sleep (50 women, 36.0%), progressively worse sleep (67 women, 48.2%), and persistently poor sleep (22 women, 15.8%). Poor sleep was significantly associated with pre-pregnancy BMI and more baseline (third-trimester pregnancy) depressive and fatigue symptoms. At 6 months post-CS, women classified as progressively worse or persistently poor sleepers showed a trend toward higher BMI (p<0.03), more depressive symptoms (p<0.001), and higher fatigue scores (p<0.001) than those with stable poor sleep.

**Conclusions:**

Women had three distinct sleep-disturbance trajectories before and after elective CS. These poor-sleep courses were associated with BMI and psychological well-being. Our findings suggest a need to continuously assess sleep quality among women considering elective CS and up to 6 months post-CS.

## Introduction

After cesarean section (CS), women appear to be at greater risk for sleep disturbance [1,2,3], but changes in their sleep patterns and characteristics over time remain unclear. This lack of information is especially marked in late pregnancy and early postpartum, which has been recognized as a period of vulnerability to sleep issues [1]. In Taiwan, the CS rate has rapidly increased from 33.9% in 2006 to 37.6% in 2012 [4]. Despite concerns about the continuous rise in CS rates worldwide as a notable public health issue, CS is generally regarded in many sleep studies as merely an obstetric variable. Furthermore, no study was found on the sleep characteristics of women considering CS or who underwent elective CS. Lack of such studies will hamper development of effective strategies to deal with the prevalent and distressing ante- and postnatal symptoms of sleep disturbance, which are known to adversely affect childbearing women’s health, well-being, and maternal role performance, such as breastfeeding [5,6].

Another gap in knowledge is that sleep patterns during pregnancy or postpartum have mostly been investigated separately, with little attention paid to childbearing as a continuous dynamic process. Cross-sectional sleep studies that focus on the prenatal phase cannot fully reflect the real status of maternal sleep changes over time and the relation between pre- and postnatal sleep. Moreover, previous research has focused on pregnant women as a uniform population rather than considering the possibility of distinctive subgroups, which might limit understanding of individual variability. Distinct patterns of symptom change over time have recently been reported in perinatal women [7,8]. Identification and characterization of meaningful trajectory clusters provide a better understanding of different patient subgroups, helping to identify which clients to target and the critical period for early interventions, two important aspects of clinical care.

Sleep characteristics of childbearing women have been associated with several factors, including maternal body weight [9,10], depression [11,12], and fatigue [13,14,15,16]. Since maternal obesity is known to be a predisposing factor for sleep disorders [17], the relationship between sleep quality and weight gain during pregnancy has attracted increasing attention. Indeed, sleep deprivation has been shown to increase the risk of postpartum weight gain [9], and gaining more weight during pregnancy has been suggested to exacerbate sleep apnea in obese women [10]. Furthermore, 148 pregnant women with high levels of depressive symptoms were shown to have significantly poorer sleep quality than those without depressive symptoms [11].

Fatigue is a major concern for many childbearing women [14]; both lay and scientific opinions deem that fatigue may compromise maternal sleep [1], but empirical evidence is inconclusive. For example, self-reported sleep disturbance scores and fatigue of 72 pairs of parents before and after childbirth were positively correlated in one study [13], but other studies found little association between sleep disruption and fatigue [18,19]. However, the above studies were limited by assessing only hours of night sleep [9,14] or sleep apnea [10], using a cross-sectional design [11], measuring sleep outcomes at only two points [13], or being outdated [18,19]. Thus, the longitudinal relationships among sleep patterns, body weight, depressive symptoms, and fatigue symptoms in childbearing women need to be clarified.

Therefore, we analyzed data from a prospective study to identify sleep-disturbance trajectories of women considering elective CS from the third trimester of pregnancy to 6 months post-CS and to examine the associations of sleep-disturbance trajectories with body mass index (BMI), depressive symptoms, and fatigue scores.

## Methods

### Participants

We analyzed data of 139 pregnant Taiwanese women in a prospective follow-up study as described [7]. In brief, pregnant women attending an antenatal care clinic for prenatal care were invited to participate if they were ≥20 years old and considering an elective CS. Women were excluded if they were experiencing high-risk conditions associated with their pregnancies (e.g., pregnancy-induced hypertension, diabetes, hemorrhage, preterm delivery, low birth weight), had any chronic medical illness (e.g., hypertension, peptic ulcer, or renal, endocrine, psychiatric, neurologic, and cardiovascular disease), or had a diagnosed sleep disorder. Participating women were first assessed in their third trimester and again at 1 day, 1 week, 1 month, and 6 months postpartum. The study protocol was approved by the institutional review board of China Medical University Hospital, Taiwan, and written informed consents were obtained from all participants.

### Measures

#### Sleep quality

Sleep quality and disturbances were assessed using the 19-item Pittsburgh Sleep Quality Index (PSQI) [20]. A global score ranging from 0 (good sleep) to 21 (very poor sleep) was computed from seven component scores (0–3): subjective sleep quality, sleep latency, sleep duration, habitual sleep efficiency, sleep disturbances, use of sleeping medications, and daytime dysfunction. Mothers were identified as having “insomnia” if they agreed with two statements: “Cannot get to sleep within 30 minutes” or “Wake up in the middle of the night or early morning.” The reliability and validity of the Taiwanese version PSQI have been well established [21]. The PSQI is widely used for detecting sleep problems in childbearing women with internal reliability (Cronbach’s alpha) of 0.73 [22]; in our study, Cronbach’s alphas ranged from 0.70 to 0.78.

#### Depressive symptoms

Depressive symptoms were measured by the 10-item Edinburgh Postnatal Depression Scale (EPDS) [23]. Responses to the EPDS are rated on a 4-point scale from 0 (no) to 3 (most of the time), with total scores ranging from 0 to 30. A higher score represents a higher level of depression. The EPDS was shown to have good psychometric properties among childbearing women, with an internal reliability of 0.87 [23]. The Taiwanese version EPDS had an internal reliability of 0.83 [24] and good validity [25]. In this study, Cronbach’s alpha of the EPDS was 0.82–0.86.

#### Fatigue symptoms

Fatigue symptoms over the previous weeks were assessed using the 30-item Fatigue Continuum Form (FCF) [26]. Responses to each question are rated on a 4-point scale from 1 (not at all) to 4 (very much so), with total scores ranging from 30 to 120. The FCF has been shown to perform well in childbearing women with good reliability and validity [26]. The FCF has been translated to Taiwanese and validated [27]. In the current study, the Cronbach’s alpha of the FCF was 0.90–0.94.

#### Body weight and height

Data on body weight and height were collected by participants’ self-report at baseline (third-trimester pregnancy) as well as 1 day, 4 weeks, and 6 months post-CS. Body mass index (BMI) was then calculated as the weight in kilograms divided by the height in meters squared (kg/m^2^).

#### Demographic measures and health status

During the first assessment, mothers reported their age, education attainment, employment status, whether or not the current pregnancy was planned, as well as physiologic variables, including parity and prenatal exercise. Medical information was also obtained from participants’ medical records, including hemoglobin levels during pregnancy as well as use of patient-controlled analgesia (PCA) and pain medication after delivery. Routine blood tests for hemoglobin levels were completed between weeks 24 and 28 of pregnancy. Hemoglobin levels < 11 g/dl were considered indicators of anemia [28].

### Statistical Analysis

To identify distinctive sleep-disturbance (PSQI scores) groups of patients over the follow-up period, we started with trajectory analyses that used semi-parametric group-based trajectory modeling [29] as applied in PROC TRAJ in SAS [30]. This analysis was person centered [29], i.e., it was based on each mother’s PSQI score from their third trimester until 6 months postpartum. This kind of analysis is designed to estimate growth curves for each individual and to classify individuals with similar growth curves into different trajectory groups. This method allows the use of data when the number of assessments varies across participants, which is common in longitudinal studies. Selection of the optimal trajectory group was based on the model with the smallest absolute Bayesian information criterion (BIC) value [29]. Mothers were classified into different sleep-disturbance trajectory groups according to the highest posterior probabilities for group membership. Participant characteristics and symptom severity scores were analyzed using descriptive statistics and frequency distributions in SAS software, version 9.2 (SAS Institute, Cary, NC, USA). Univariate associations between sleep trajectories and baseline characteristics were examined using χ^2^ and one-way ANOVA tests. Multivariate relationships between membership in sleep-trajectories groups and baseline variables were examined using logistic regression analysis. To examine the relationships between sleep-trajectory membership and subsequent BMI, depressive symptoms, and fatigue symptoms, we used analysis of covariance with adjustment for covariates and the trend test. We calculated the effect size as the standardized difference between two means divided by the pooled standard deviation of each group [31].

## Results

All participants completed four assessments from the third trimester to 1 month postpartum, and 102 (73%) completed the fifth assessment at the 6-month postpartum follow-up. Participants who completed all five assessments and those who dropped out at 6 months postpartum did not differ significantly in their mean PSQI scores at the first, second, third, or fourth assessment (*p* = 0.19–0.90). These two groups also did not differ in demographic characteristics, including age, parity, educational level, prenatal employment, and prenatal exercise (*p* = 0.14–0.90).

### Sleep-Quality Trajectory Groups

**Fig 1:**
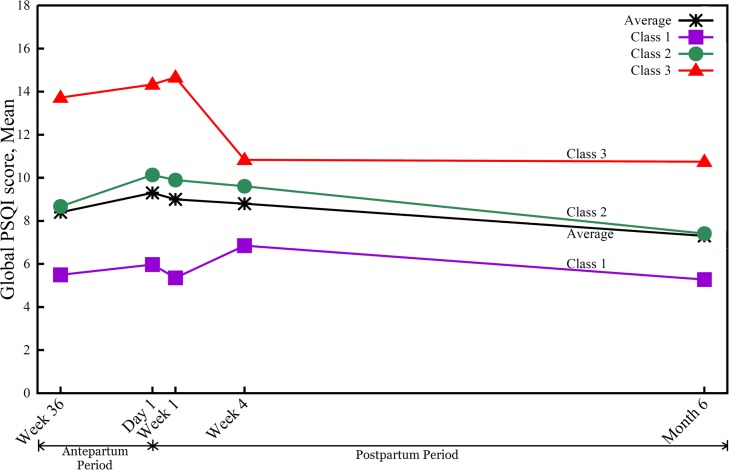
Three classes of sleep trajectories from third-trimester pregnancy to 6 months after cesarean delivery (*N* = 139). Class 1 (stable poor sleep; 36.0%), class 2 (progressively worse sleep; 48.2%), and class 3 (persistently poor sleep; 15.8%).

We identified the best fitting models, based on the lowest absolute BIC (-1688.6), for three sleep-disturbance trajectory groups: 36% of mothers (*n* = 50) followed a stable poor sleep trajectory, 48.2% (*n* = 67) were in a progressively worse sleep trajectory, and 15.8% (*n* = 22) followed a persistently poor sleep trajectory (Fig 1). Further support for adequate model fitting came from the relatively high average posterior probability for sleep trajectories, ranging from 0.89 (standard error [SE] = 0.13) to 0.92 (SE = 0.12), which were greater than the suggested criterion of 0.70 [32].

Mothers in the stable poor sleep trajectory group (class 1) had relatively low PSQI scores from late pregnancy to early postpartum (mean scores of 5.4, 5.9, and 5.2 at 36 weeks of pregnancy, 1 day post-CS, and 1 week post-CS, respectively) with a peak at 1 month postpartum (PSQI score of 6.7) and decrease at 6 months postpartum (PSQI score of 5.2). For the progressively worse sleep trajectory group (class 2), mothers started with a middle-high PSQI score in late pregnancy (score of 8.9), peaked at 1 day postpartum (score of 10.2), and then gradually decreased but remained at a middle-high PSQI score at 6 months postpartum (score of 7.6). Mothers who reported the worst subjective sleep quality (class 3) started with the highest PSQI score (14.1) in late pregnancy, increased to 14.7 at 1 week postpartum, with a sharp decrease at 1 month postpartum (score of 11.0) but maintained a high score at 6 months postpartum (score of 10.7). The total PSQI scores for all three trajectories were > 5, with scores for classes 2 and 3 > 8, indicating sleep disturbance based on the commonly suggested cutoff of 5 [20].

Taken together, the average PSQI scores for all three groups of women were poor (Fig 1), starting at PSQI of 8.4 in late pregnancy, increasing to 9.3 and 9.0 at 1 day and 1 week postpartum, respectively, and slowly decreasing to 8.8 and 7.3 at 1 month and 6 months, respectively. The global pattern of sleep quality remained poor across postpartum and slowly returned at 6 months postpartum to the late-pregnancy level.

**Fig 2:**
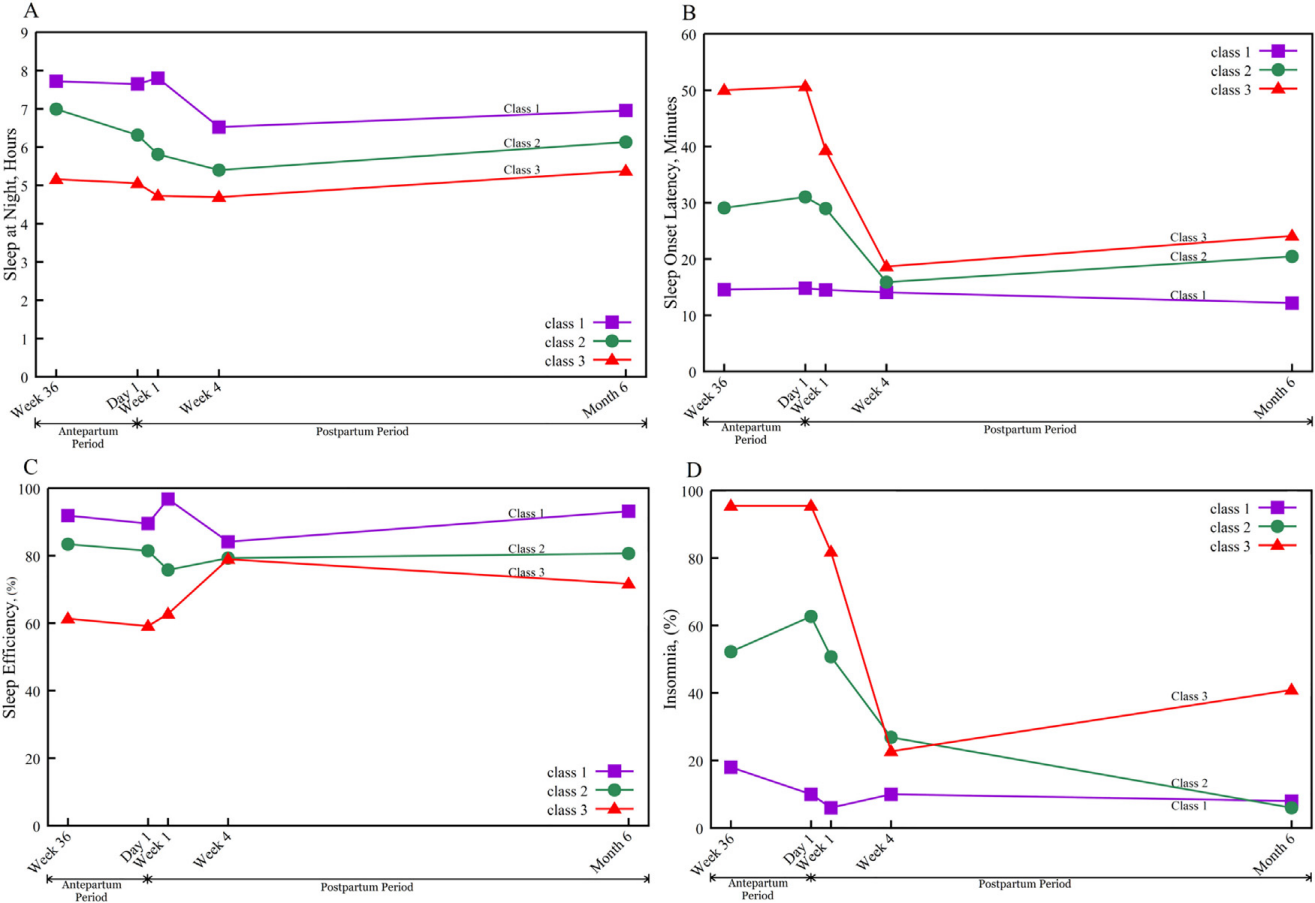
Trajectories of sleep components from third-trimester pregnancy to 6 months post-cesarean delivery (*N* = 139). **A:** sleep at night (hours); **B:** sleep onset latency (minutes); **C:** sleep efficiency (%), and **D:** insomnia (%).

The trajectories of the PSQI sleep components for the three classes are displayed in Fig 2A through 2D, showing the time courses for amount of night sleep (hours), sleep onset latency (minutes), night sleep efficiency, and participants with insomnia (percent), respectively.

### Baseline Characteristics Associated with Sleep Trajectories

Participants’ baseline characteristics (at 36 weeks pregnancy) are shown in Table 1. They had a mean age of 33.6 years (standard deviation [SD] = 3.8; range = 24–42), with the majority (77.8%) over 30 years old. The majority of participants were multipara (61%), had at least some college education (73.4%), and were employed at study entry (64%). The majority reported not engaging in prenatal exercises (64%) and that their pregnancy was planned (66.2%). About one-third of participants had anemia during pregnancy (33.8%), and after CS, most of them used PCA (85.6%) and took pain medications (99.7%). The mean gestational age at delivery was 37.3 weeks (SD = 2.0), and the newborns’ average birth weight was 3363.4 g (SD = 678.3). Demographic variables (age, parity, education, and prenatal exercise) were not significantly associated with any sleep trajectory. Furthermore, anemia during pregnancy, PCA use after CS, and pain medication use after CS did not differ among the three sleep groups.

**Table 1:** Comparison of baseline characteristics among sleep trajectories classes (n = 139).

		Class 1	Class 2	Class 3		
Variable	All	Stable Poor Sleep	Progressively Worse Sleep	Persistently Poor Sleep	χ^2^/F	*P*
	(n = 139)	(n = 50)	(n = 67)	(n = 22)		
	n(%)	n(%)	n(%)	n(%)		
Age (years)					0.16	0.90 ^a^
≤30	31(22.3)	12(24.0)	14(20.9)	5(22.7)		
>30	108(77.8)	38(76.0)	53(79.1)	17(77.3)		
Parity					1.58	0.48
Primiparas	53(38.1)	16(32.0)	29(43.3)	8(36.4)		
Multiparas	86(61.9)	34(68.0)	38(56.7)	14(63.6)		
Education					0.95	0.68 ^a^
≤ High school	37(26.6)	14(28.0)	19(28.4)	4(18.2)		
≥ College	102(73.4)	36(72.0)	48(71.6)	18(81.8)		
Employment					0.63	0.72
No	50(36.0)	16(32.0)	25(37.3)	9(40.9)		
Yes	89(64.0)	34(68.0)	42(62.7)	13(59.1)		
Prenatal exercise					1.02	0.60
No	89(64.0)	33(66.0)	44(65.7)	12(54.6)		
Yes	50(36.0)	17(34.0)	23(34.3)	10(45.4)		
Planned Pregnancy					0.35	0.85
No	47(33.8)	18(36.0)	21(31.3)	8(36.4)		
Yes	92(66.2)	32(64.0)	46(68.7)	14(63.6)		
Anemia					2.09	0.37 ^a^
No	92(66.2)	30(60.0)	45(67.2)	17(77.3)		
Yes	47(33.8)	20(40.0)	22(32.8)	5(22.7)		
Use of PCA					1.41	0.58 ^a^
No	20(14.4)	6(12.0)	12(17.9)	2 (9.1)		
Yes	119(85.6)	44(88.0)	55(82.1)	20(90.9)		
Use of pain medication					2.93	0.38 ^a^
No	4(3.0)	3(6.2)	1(1.5)	0 (0.0)		
Yes	131(97.0)	45(93.8)	64(98.5)	22(100)		
Pre-pregnancy BMI (kg/m2)					**4.9**	0.009
Mean (SD)	22.4(4.0)	22.1(3.9)	22.1(3.4)	25.0(5.2)		
EPDS					**8.9**	<0.001
Mean (SD)	9.7(5.1)	7.6(5.0)	10.5(4.9)	12.3(4.1)		
FCF score					**17.2**	<0.001
Mean (SD)	51.3(12.0)	45.7(9.8)	52.1(10.8)	61.8(13.0)		

^a^ Fisher’s exact test

PCA, Patient-controlled analgesia; BMI, body mass index; EPDS, Edinburgh Postnatal Depression Scale; FCF, Fatigue Continuum Form.

Overall, participants’ mean pre-pregnancy BMI was 22.4 kg/m^2^ (SD = 4.0). Women in the persistently poor sleep group (class 3) had the highest BMI of all three groups; their BMI of 25 kg/m^2^ (SD = 5.2) was higher than those of women in the stable poor sleep (class 1) or progressively worse sleep group (class 2) (p = 0.009). The total sample mean scores for depressive symptoms (EPDS scores) and fatigue (FCF scores) were 9.7 (SD = 5.1) and 51.3 (SD = 12.0), respectively. The progressively worse sleep group and persistently poor sleep group had significantly higher mean EPDS and FCF scores (p = 0.0002 and p = 0.0001, respectively), than the stable poor sleep group. Multivariate associations were examined between baseline variables and sleep trajectory class by logistic regression analyses after adjusting for significantly different variables in Table 1, using stable poor sleep (class 1) as the reference category and comparing it to the combined progressively worse and persistently poor trajectories. The results of multivariate analyses showed that mothers with high levels of depressive symptoms (EPDS scores ≥12) (odds ratio [OR] = 3.8, 95% confidence interval [CI] = 1.3–11.1, *p* < 0.01) or fatigue symptoms (FCF scores ≥51) (OR = 3.0, 95% CI = 1.3–6.9, *p* < 0.01) were more likely to be in the progressively worse or persistently poor trajectory. Demographic variables were not significantly associated in multivariate analyses with pre-pregnancy BMI for the progressively worse or persistently poor trajectories (data not shown).

### PSQI Component Scores and Sleep Variables

As shown in Table 2, the mean total PSQI score was 8.4 (SD = 3.7) in late pregnancy, then gradually increased to 9.3 (SD = 3.8) and 9.0 (SD = 4.2) at 1 day and 1 week postpartum, respectively. The PSQI score decreased to 8.8 (SD = 3.1) at 4 weeks postpartum, reaching the lowest score of 7.3 (SD = 3.5) at 6 months postpartum. Most PSQI component scores at each assessment time were >1, reflecting disturbed sleep as suggested [18]. However, PSQI scores for sleep medication were < 0.02 across the five assessments, indicating Taiwanese women used little sleep medication before and after CS delivery.

**Table 2:** PSQI component scores and sleep variables at each assessment.

	36 weeks of pregnancy	1 day postpartum	1 week postpartum	4 weeks postpartum	6 months postpartum
	(n = 139)	(n = 139)	(n = 139)	(n = 139)	(n = 102)
Component score (0–3)	Mean (SD)	Mean (SD)	Mean (SD)	Mean (SD)	Mean (SD)
PSQI total (0–21)	8.4 (3.7)	9.3 (3.8)	9.0 (4.2)	8.8 (3.1)	7.3 (3.5)
Subjective sleep quality	1.7 (0.8)	1.9(0.8)	1.8 (0.8)	1.9 (0.8)	1.4 (0.9)
Sleep latency	1.5 (0.9)	1.6 (0.9)	1.4 (0.9)	1.0 (0.8)	1.1 (0.8)
Sleep duration	0.7 (0.9)	1.0 (1.0)	1.1 (1.1)	1.5 (1.1)	1.0 (1.0)
Sleep efficiency	1.0(1.1)	1.2 (1.2)	1.2 (1.2)	1.2 (1.3)	0.9 (1.1)
Sleep disturbances	1.8 (0.6)	1.8 (0.6)	1.6 (0.6)	1.2 (0.6)	1.1 (0.6)
Sleep medication	0.14 (0.6)	0.14 (0.6)	0.13 (0.5)	0.05 (0.3)	0.09 (0.4)
Daytime function	1.6 (0.8)	1.7 (0.8)	1.7 (0.9)	2.0 (0.9)	1.6 (0.9)
Sleep variables, by PSQI					
Sleep onset latency, minutes	27 (23)	28 (22)	25 (19)	15 (13)	18 (14)
Sleep at night, hours	7.0 (1.6)	6.6 (1.9)	6.4 (2.0)	5.7 (1.8)	6.3 (1.5)
Sleep efficiency, %	83 (19)	80 (23)	81 (26)	81 (29)	83 (21)
Insomnia, n (%)	65 (47)	69 (49)	55 (40)	28 (20)	17 (12)

PSQI, Pittsburgh Sleep Quality Index.

The sleep variable results indicate that mothers spent an average of 25–28 minutes to fall asleep (sleep onset latency) from late pregnancy to 1 week postpartum, with less time needed (15–18 minutes) after 4 weeks postpartum. The women slept more hours at night, on average, during pregnancy (7 hours) and the fewest hours (5.7) at 4 weeks postpartum with a slight increase to 6.3 hours at 6 months postpartum. The mean sleep efficiency was below 85% across the five assessments, ranging from 80% to 83%. The prevalence of insomnia was assessed as the percentage of women with at least one insomnia symptom (“Cannot get to sleep within 30 minutes” or “Wake up in the middle of the night or early morning”). The prevalence rate of insomnia was 47%, 49%, 40%, 20%, and 12% in the third trimester of pregnancy, 1 day, 1 week, 4 weeks, and 6 months postpartum, respectively, suggesting that the insomnia rate decreased as women approached 6 months postpartum.

### Sleep Trajectories and Body weight, Depression, and Fatigue

BMI at 4 weeks (p = 0.06) and 6 months (p = 0.07) postpartum differed among the three sleep trajectory groups at borderline significance (Table 3). Mothers with persistently poor sleep trended toward a higher BMI at 6 months postpartum (p = 0.03). At 4 weeks or 6 months postpartum, mothers with progressively worse or persistently poor sleep tended to have more depressive symptoms (p <0.01) or greater fatigue (p <0.01), with effect sizes ranging from 0.53–0.95 for depressive symptoms and 0.48–1.28 for fatigue symptoms, respectively.

**Table 3:** BMI, depressive symptoms, and fatigue scores at postpartum follow-ups by sleep trajectory class.

	Class 1	Class 2		Class 3				
	Stable Poor Sleep	Progressively Worse Sleep		Persistently Poor Sleep		Group comparison (ANCOVA)^b^		Trend test^c^
	Mean (SD)	Mean (SD)	ES^a^	Mean (SD)	ES^a^	F (df)	*P*	*P*
BMI								
4 weeks	24.9 (4.1)	24.5 (3.6)	-0.10	27.1 (4.5)	0.51	2.81 (2, 90)	0.06	0.12
6 months	22.6 (4.0)	23.3 (3.9)	0.18	25.5 (5.8)	0.58	2.07 (2, 94)	0.07	0.03
EPDS								
4 weeks	6.9 (5.6)	10.0 (6.0)	0.53	10.4 (6.1)	0.60	4.76 (2,134)	0.01	0.01
6 months	5.5 (5.0)	8.5 (5.6)	0.57	10.0 (4.4)	0.95	6.00 (2, 96)	0.003	0.001
FCF								
4 weeks	42.1 (8.0)	50.0 (12.7)	0.74	58.5 (16.4)	1.27	15.3 (2,134)	0.001	0.001
6 months	42.0 (9.9)	47.1 (11.5)	0.48	55.8 (11.6)	1.28	10.3 (2, 96)	0.001	0.001

^a^ Effect size, based on comparison with class 1.

^b^ All analyses were adjusted for baseline age and parity.

^c^ Linear regression with class 1 coded as 1, class 2 coded as 2, and class 3 coded as 3.

BMI, body mass index; EPDS, Edinburgh Postnatal Depression Scale; FCF, Fatigue Continuum Form.

## Discussion

Our analysis of prospective study data characterized the sleep quality of women who considered CS from late pregnancy to the early motherhood period, a high-risk time for sleep disturbance [33] and identified three distinct sleep quality trajectories. All three sleep trajectories were characterized by mean PQSI scores > 5, with two sleep trajectories representing nearly two-thirds of the women having mean PQSI scores > 8. These results show that the majority of these women had sleep disturbances until 6 months postpartum. The mean sleep quality scores of our participants were higher than those of pregnant and postpartum women in most studies [5,12,16,22], but similar to those of 51 non-depressed mothers with a history of postpartum major depression [6]. One reason for the difference in PQSI scores may be that most participants in previous studies had vaginal deliveries [5]. The delivery process in CS is very different from vaginal delivery; CS mothers have to go through surgery and wound pain, which may lead to more sleep problems than after vaginal delivery. The effect of delivery mode on mothers’ sleep has not been well studied. In one preliminary study of mothers’ sleep patterns while their newborn infants were hospitalized in the intensive care unit, six hospitalized CS mothers had considerably worse sleep than 15 mothers discharged after vaginal delivery and sleeping at home [2]. Another possible reason for the higher PSQI scores in our sample of CS mothers is fear of childbirth, which has been reported as a factor contributing to elective CS [34]. This possibility is supported by a report that childbirth fear was positively correlated with sleep deprivation in a cross-sectional descriptive survey of 650 community-dwelling pregnant women [15]. A retrospective population-based cohort study in Finland has shown that fear of childbirth and CS birth were predisposing risk factors for postpartum depression [35]. Given the worldwide trend towards increasing CS rates and the adverse effect of sleep disturbance on maternal and infant health [36,37], health care providers should pay more attention to the sleep problems of women after CS.

We measured sleep quality using the PSQI, which has been widely used in various clinical populations and validated in pregnant women [6,11,38]. The criterion for distinguishing between good and poor sleep quality in general is a total PSQI score ≥5 [20], and the same PSQI cutoff score has been used for sleep quality in pregnancy and early postpartum [12,22,36]. Using the same PSQI cutoff score for pregnant women is questionable since most studies consistently show that the great majority of women suffer from sleep disturbance through pregnancy to postpartum, making it difficult to distinguish groups at high risk for sleep disturbance [5,12,39]. When sleep quality was compared in depressed and non-depressed mothers between 13 and 20 days postpartum, all depressed mothers and 90% of non-depressed ones were poor sleepers when the PSQI cutoff score was ≥ 5; thus, the authors concluded that global PSQI scores of postpartum mothers were similar to those of depressed patients [40]. In an early study on the psychometric properties of the PSQI in four different clinical populations [41], all groups with sleep problems were reported to have mean PSQI scores greater than 8.0 although the data were not shown. The authors suggested that a more suitable criterion for poor sleep quality would be a cutoff score of 8.0 [41].

More recently, the association between sleep quality and recurrence of postpartum depression was shown to be significant with a PSQI cutoff score of d 5 [6]. Although the authors suggested changing the PSQI cutoff to >7 for pregnant women who differ from non-pregnant women in having higher PSQI scores, more sleep complaints, and using less sleep medicine, they did not do so because they lacked empirical evidence [6].

In our study, we did not use an arbitrary PSQI cutoff score, but adopted an individual-based analysis. This method estimated each mother’s sleep curve over time and categorized these changes into different sleep trajectories, with sufficient evidence to warrant both an appropriate cut point and categorization.

Our results on the natural evolution and characteristics of sleep trajectories of CS mothers show that those who suffered poorer sleep quality in the third trimester of pregnancy also had disturbed sleep after birth. This finding implies the third trimester is a critical time for early intervention. As gestation advances, physical and psychological discomforts usually gradually increase, such as frequent micturition, low back pain, leg cramps, and mood disturbance [42]. These discomforts have been well recognized to be associated with more sleep disruption in late pregnancy [42,43]. In line with a previous report [16], we suggest routine screening for maternal sleep quality during prenatal checkups, especially the third trimester.

Another critical time point for assessing sleep quality is 1 month postpartum. All women with CS in our study sample, even those in the stable poor-sleep trajectory group, reported their highest scores for subjective sleep quality, sleep duration, and daytime function in the first month postpartum. Our results (Table 2) show that all mothers had impaired sleep efficiency (80–83%) across five measurement points. However, after categorization, mothers in the stable poor-sleep trajectory group had good sleep efficacy except 1 month after birth.

The first month postpartum is a period encompassing considerable physiological and psychological changes for CS mothers; during this month, they have to recover after surgery, adapt to the new maternal role, and assume responsibility for infant care [40]. Thus, both late-trimester pregnancy and the first month postpartum are periods of concern about sleep hygiene. These findings also support the importance of longitudinal follow-up and categorization of sleep-quality trajectories to thoroughly describe the dynamic phenomenon of maternal sleep, thus contributing more information for clinical practice.

We found that most scores for the seven PQSI components at different times were >1, suggesting disturbed sleep [20]. This finding echoes previous evidence that 400 Taiwanese third-trimester pregnant women had five PQSI component scores > 1 [16]. We also found that the persistently poor sleep trajectory group had the highest PQSI component scores among the three groups. This result was not surprising since this group had longest sleep onset latency, slept fewer hours at night, and had lower sleep efficiency. Very few mothers in each group used sleep medication, consistent with a prior report that pregnant Taiwanese women rarely used medication [22]. In our study, most of the women using sleep medication were also in the persistently poor sleep trajectory group. Our results (Fig 2D) also show the rate of insomnia peaked in late pregnancy or the first day postpartum among all three classes, consistent with reports that late pregnancy is a high-risk period of sleep disturbance [44,45]. More women in the progressively worse and persistently poor sleep groups suffered from insomnia than in the stable poor sleep group, and the incidence of insomnia decreased in all groups with time. Of note, 40% of the women in the persistently poor sleep group suffered from insomnia at 6 months postpartum, once again confirming that this group is at high risk for sleep problems. Insomnia may contribute to an individual’s psychological well-being, underscoring the importance of further studies on the influence of insomnia on childbearing women [46].

Our results show bidirectional relationships among sleep, body weight, depression, and fatigue. Women with higher BMI levels, more depressive symptoms, and more fatigue were more likely to belong to the middle or high poor-sleep groups. Similarly, these women also reported significantly higher BMI, more depressive symptoms, and more fatigue at 1 and 6 months postpartum. Our results are consistent with a report that 940 women with shorter sleep duration at 6 months postpartum were more likely to retain ≥ 5 kg above their pre-pregnancy weight at l year postpartum [9]. In that study, however, sleep quality was not measured. No studies have explored the relation between sleep quality and weight gain during the childbirth period. Furthermore, an intensive systematic review failed to find any consistent reports of a relationship between sleep duration and subsequent weight retention in adults [47]. Overweight and obesity are known to threaten women’s health; maternal weight gain during pregnancy is regarded as an important cause of weight problems after childbirth [48]. Thus, identifying risk factors for weight retention in pregnancy will contribute to curbing the obesity rate. More studies are suggested to continue evaluating the relationship between sleep and BMI among childbearing women.

The close bidirectional causal relationship between sleep and mood may derive from their common neurobiological and physiological foundations [49]. Indeed, a growing body of evidence illustrates that depressive symptoms are significantly associated with sleep disturbance among childbearing women [12,33,50]. Similarly, sleep disturbance has been hypothesized to be a modifiable risk factor for developing or recurring depression, although the results remain inconclusive [49]. In one longitudinal study [51], subjective measures of poor sleep were more accurate predictors of postpartum depressive symptoms than objective sleep measures, whereas another study found no evidence that depressive symptoms in early pregnancy affected sleep quality at later stages of pregnancy [52]. These different results may be affected by the complex relationship between sleep quality and depression [11]. Indeed, a prospective study of the relationships among sleep quality, physical symptoms and depressive symptoms in pregnancy found that poor sleep quality mediated the association of second-trimester physical symptoms and third-trimester depressive symptoms [38]. The authors emphasized the importance of screening for poor sleep quality in late-stage pregnancy to begin early management of postpartum depression, a suggestion supported by our findings.

Our findings on sleep quality, depression, and fatigue are not only consistent with prior cross-sectional [51,53] and longitudinal studies [54], but also extend that knowledge by identifying different poor sleep-quality trajectories to verify the bidirectional causal relationship among these three variables. For example, fatigue and poor sleep have been positively correlated in pregnant women [15,16] and are common complaints among new mothers, particularly those with mood disturbance [51,53]. Furthermore, a bidirectional relationship was found among sleep quality, fatigue, and depression in a repeated-measures study; fatigue and depression predicted sleep quality, and sleep quality was a predictor of fatigue and depression [54].

This study had a few potential limitations. First, we did not assess participants for pregnancy-related sleep disorders such as obstructive sleep apnea (OSA) and restless legs syndrome (RLS). Since OSA and RLS have been associated with a greater risk of depression during pregnancy [55] and in postpartum [56], respectively, not assessing these conditions may have affected our results on depression. However, this effect may have been minimized by our participants not reporting any diagnosed sleep disorders before and after pregnancy and our excluding women with several risk factors for OSA or RLS, including hypertension, thyroid disease, and peptic ulcer [57]. Furthermore, no participants had perinatal complications that have been associated with OSA, such as preeclampsia and preterm or low birth weight infants [58]. Future perinatal sleep studies are suggested to take into account participants’ OSA and RLS. Second, the lack of objective sleep measures, such as actigraphy may have limited the validity of the results. However, sleep quality is highly subjective, and the accessibility and convenience of subjective self-reports of sleep quality have irreplaceable clinical value. Besides, perceived sleep quality (PSQI scores) has been shown in pre- and postnatal longitudinal studies to be more strongly associated with postpartum mood than objective measures [5,33,36,44]. Third, we used the PSQI to collect sleep data rather than a subjective scale assessing a single night of sleep, which would have been more appropriate. However, the PSQI was shown to have good 2-day and 45-day test–retest reliabilities of 0.90 and 0.86, respectively, in patients with primary insomnia [59]. Furthermore, the PSQI has been widely used to assess sleep quality in women during pregnancy and postpartum [6,12,16,22,33,36,43]. Fourth, our participants elected to undergo CS, possibly limiting the generalizability of the findings to the entire population of CS mothers. We suggest that future research increase sample heterogeneity by including high-risk or emergency CS cases and enhance comparison of sleep trajectories by including women who gave birth by both CS and vaginal delivery. Finally, we did not collect data on participants’ vitamin intake during pregnancy. Consumption of vitamins and minerals such as iron or folic acid has been suggested to be related to RLS during pregnancy [60].

In conclusion, by longitudinally tracing the sleep quality of women who planned to undergo elective CS, we identified three distinct sleep trajectories. We found that all participants in the three trajectory groups suffered from poor sleep quality, especially those who had worse sleep quality in the third trimester. Furthermore, our findings show that the courses of poor sleep were associated with maternal body weight and psychological well-being. To the best of our knowledge, this study is the first to differentiate different patterns of sleep quality over time, to describe the discrepancies in sleep components among different sleep-trajectory groups, and to identify a bidirectional relationship among sleep, body weight, and mood that is correlated with different sleep trajectories. These finding have particularly important clinical implications because they contribute to better understanding the critical timing for early preventive interventions, to identifying the high-risk groups to target for intervention, and provide new insights into interventions for managing postpartum depression.
